# Partial N-gene target failure in the Seegene Allplex SARS-CoV-2 Master Assay as a proxy of SARS-CoV-2 BA.2.86

**DOI:** 10.1128/spectrum.00179-24

**Published:** 2024-04-22

**Authors:** Maria Beatrice Valli, Maria Letizia Schiavone, Martina Rueca, Giulia Berno, Pietro Giorgio Spezia, Cesare Ernesto Maria Gruber, Federica Forbici, Lavinia Fabeni, Daniele Focosi, Enrico Girardi, Marcello Meledandri, Fabrizio Maggi

**Affiliations:** 1 Laboratory of Virology, National Institute for Infectious Diseases, Lazzaro Spallanzani IRCCS, Rome, Italy; 2 UOC Microbiology and Virology, San Filippo Neri Hospital, ASL Roma 1, Rome, Italy; 3 North-Western Tuscany Blood Bank, Pisa University Hospital, Pisa, Italy; 4 Scientific Direction, National Institute for Infectious Diseases, Lazzaro Spallanzani IRCCS, Rome, Italy; University of Siena, Siena, Italy

**Keywords:** Seegene Allplex, SARS-CoV-2, PCR, partial target failure, nucleocapsid, BA.2.86

## LETTER

More than 1,000 types of molecular nucleic acid amplification tests (NAAT) and antigen-based tests to detect SARS-CoV-2 are now available worldwide (1). During the COVID-19 pandemic, mutations that determined NAAT target failures have been used to identify SARS-CoV-2 variants, with epidemiological implications.

The most notable example of target failure has been the 6-nucleotide deletion (21765–21770, leading to amino acid Δ69–70HV) in the spike gene of Alpha and Omicron (B.1.1.529, BA.1, BA.4, and BA.5 variant of concern (VOCs) and the BA.2.86* variant of interest (VOI), which causes S-gene target failure on a widely used commercial test, the 3-target TaqPath RT-PCR COVID-19 assay (Thermo Fisher Scientific) (2, 3). The kit also suffered from N-gene target failure (NGTF) with several Delta polymorphisms and has since been updated (4). Several cases of NGTF have also been reported over time in the setting of commercial assays (5–8). Among them, NGTF [Δ6–10, N-gene dropout on cycle threshold (Ct) values >29] has been previously proven specific for Alpha-positive samples using the Allplex SARS-CoV-2/FluA/FluB/RSV PCR assay (9). The N:641∆6 mutation in AY.4 (resulting in a two-amino acid deletion of G214 and G215) has been similarly shown to cause NGTF in the Allplex SARS-CoV-2 assay(10).

In November 2023, while performing diagnostic PCRs, we noted a reduced amplification of the SARS-CoV-2 N gene compared to the other targets in two nasopharyngeal swab (NPS) samples tested with the Seegene Allplex SARS-CoV-2 Master Assay. This assay targets three genes (E, RdRp/S, and N) and five mutations in the S gene (Δ69–70HV, delY144, E484K, N501Y, and P681H) (11). We later confirmed these samples as BA.2.86* by whole genome sequencing. Notably, BA.2.86* includes a novel mutation within the N gene, N:Q229K, which falls into the leucine-rich sequence (218–231) within one of the flexible intrinsically disordered regions recently shown to be a N protein oligomerization site (12).

To further investigate this preliminary finding, we tested 24 more NPS (for a total of 26) characterized as BA.2.86* in the context of national SARS-CoV-2 genomic surveillance. As shown in Table 1, we found a partial failure in N gene amplification in 25 out of 26 samples, with a reduction range of 6–12 Ct’s in the 16 samples quantifiable and with a Ct <30. Only one sample (#17) did not exhibit reduced amplification; of note, it had the highest Ct value. However, additional NGS investigations revealed the presence of an infecting SARS-CoV-2 minoritarian variant (about 20% relative abundance) characterized by the absence of the Q229K mutation for sample #17 while the other NPS tested presented the N:Q229K substitution at 100% frequency. Given the current massive emergence of BA.2.86*, particularly the JN.1 sublineage (13), the Seegene Allplex SARS-CoV-2 Master Assay can help with the early identification of JN.1, providing a cost-effective alternative to monitor its prevalence while competing against the FLip (S:L455F+S:F456L) sublineages (14).

**TABLE 1:** Results of BA.2.86* samples by Seegene Allplex SARS-CoV-2 Master Assay^*a*^

Sample	SARS-CoV-2 lineage	SARS CoV-2 gene targeted by Seegene Allplex SARS-CoV-2 Master Assay (Ct)^*b*^		
		E	RdRP/S	N
#1	JN.1	26.2	27.3	38.8
#2	BA.2.86.1	24.4	25.4	37.4
#3	JN.1	27.5	28.8	38.5
#4	BA.2.86.1	29.8	30.9	N/A
#5	BA.2.86.1	23.1	23.7	36.1
#6	JN.1	37.4	37.1	N/A
#7	JN.1	29.6	30.7	N/A
#8	JN.1	30.7	31.1	N/A
#9	BA.2.86.1	29.6	30.6	N/A
#10	BA.2.86.3	23.3	24.2	36.9
#11	JN.1	24.6	25.5	N/A
#12	JN.1	28.6	30.0	N/A
#13	BA.2.86	28.6	30.5	38.2
#14	JN.1	23.3	24.6	34.1
#15	JN.1	18.5	19.7	30.0
#16	JN.1	21.7	23.3	32.7
#17	JN.1	35,4	40.0	38.2
#18	JN.1	23.5	24.9	34.7
#19	JN.1	23.4	24.9	30.9
#20	JN.2	29.6	31.8	N/A
#21	JN.2	27.3	28.5	35.2
#22	JN.1	20.7	22.0	29.4
#23	BA.2.86	28.5	30.9	N/A
#24	JN.1	21.2	22.6	32.8
#25	JN.1	20.7	21.4	30.0
#26	BA.2.86	21.4	22.2	33.9

^*a*^ Abbreviation: N/A, not applicable for negative result.

^*b*^ S variant target results are not shown. The original purpose of this target was to identify S mutations (Δ69-70HV, delY144, E484K, del. Y144, N501Y, and P681H) for characterizing SARS-CoV-2 lineages no longer circulating in the Omicron era.
